# Abrupt shift in the observed runoff from the southwestern Greenland ice sheet

**DOI:** 10.1126/sciadv.1701169

**Published:** 2017-12-13

**Authors:** Andreas P. Ahlstrøm, Dorthe Petersen, Peter L. Langen, Michele Citterio, Jason E. Box

**Affiliations:** 1 Department of Glaciology and Climate, Geological Survey of Denmark and Greenland, Copenhagen, Denmark.; 2 Department of Hydrology, Climate and Environment, Asiaq Greenland Survey, Nuuk, Greenland.; 3 Danish Meteorological Institute, Copenhagen, Denmark.

## Abstract

The recent decades of accelerating mass loss of the Greenland ice sheet have arisen from an increase in both surface meltwater runoff and ice flow discharge from tidewater glaciers. Despite the role of the Greenland ice sheet as the dominant individual cryospheric contributor to sea level rise in recent decades, no observational record of its mass loss spans the 30-year period needed to assess its climatological state. We present for the first time a 40-year (1975–2014) time series of observed meltwater discharge from a >6500-km^2^ catchment of the southwestern Greenland ice sheet. We find that an abrupt 80% increase in runoff occurring between the 1976–2002 and 2003–2014 periods is due to a shift in atmospheric circulation, with meridional exchange events occurring more frequently over Greenland, establishing the first observation-based connection between ice sheet runoff and climate change.

## Teaser

We find an abrupt 80% increase in observed Greenland ice sheet runoff in 2003 to be related to atmospheric circulation change.

## INTRODUCTION

**Fig. 1:**
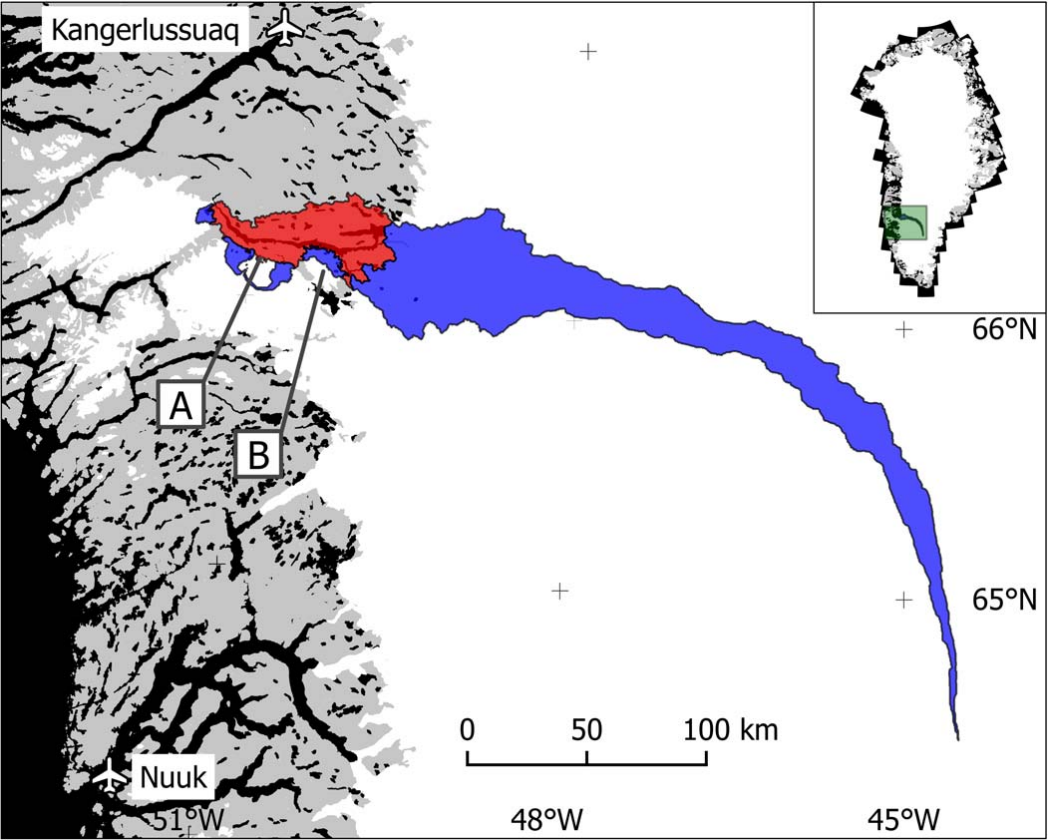
The Tasersiaq catchment in southwest Greenland. Red, ice-free part of the catchment; blue, ice-covered part of the catchment; A, unnamed ice-dammed lake delivering glacial lake outburst floods (GLOFs) to the catchment (subtracted from the discharge time series); B, Amitsulôq ice cap. Inset: map location in Greenland shown in green. A more detailed map of the outlet region is provided in fig. S1.

Quantifying the magnitude and variability of meltwater runoff from the Greenland ice sheet has until now only been possible to address through modeling (*1*, *2*). Although a new comprehensive database of in situ surface mass balance measurements has recently become available (*3*), it has not been possible to constrain the modeled runoff with in situ observations over significant temporal domains. Instead, runoff has been estimated using regional climate models forced with reanalysis data sets (*4*–*6*) or from remotely sensed data (*7*, *8*). Although modeling remains the only feasible method to estimate total runoff for the entire ice sheet, regional estimates of runoff based on in situ observations can be obtained from discharge gauges provided that the catchment can be considered spatially representative with runoff dominated by ice sheet meltwater. Here, we present a discharge time series spanning 40 years (1975–2014) from a catchment adjoining a ca. 35-km swath of the ice sheet margin, the proglacial section feeding the 65-km-long lake Tasersiaq in southwest Greenland at 66.5°N (Fig. 1 and fig. S1). The catchment is located on the northern side of the Tasersiap Sermia westward extension of the ice sheet and several local ice caps including Sukkertoppen, reaching an elevation of 2000 m above sea level (masl) and extending to the coast. Air masses passing over this barrier are depleted of moisture, implying that the Tasersiaq catchment is dominated by meltwater from the ice sheet (*9*). In the following, we partition the observed discharge in ice sheet meltwater and precipitation in the ice-free part of the catchment, respectively. This allows us to establish a 40-year time series of regional ice sheet runoff and evaluate the response to external forcing mechanisms.

## RESULTS

The Tasersiaq discharge time series, derived from a stage-discharge relationship (see Materials and Methods and fig. S2), begins on 7 July 1975 and ends on 31 October 2014, spanning 40 years with gaps of varying duration in the data coverage. Minor gaps due to maintenance visits or periods of low battery voltage (up to 7 days) were filled using linear interpolation. In total, less than 0.57% of the time series (less than 0.19% of the discharge) used for establishing annual sums was derived from interpolated values. More extensive gaps occur during freeze-in of instrumentation (during winter periods in 1975–1979) or because of other technical failures.

To determine the origin of the meltwater part of the discharge time series, we made a D-infinity delineation of the catchment based on surface slope analysis, after filling up depressions, using the Greenland Ice Mapping Project elevation model (*10*) regridded to 150-m posting (*11*). Because the large-scale drainage pattern is likely governed mainly by the basal water pressure field (*12*), the optimal delineation of the catchment should theoretically rely on knowledge of the basal topography (*13*) and water pressure, as previously attempted (*14*). However, even the most recent basal topography data set (*13*) is not of adequate resolution for this purpose, and consequentially, delineation turns out to be rather sensitive to the type of data source, as was the case in a previous study (*14*) (see discussion in the Supplementary Materials).

Using instead a terrain model for ice sheet catchment delineation is justified by earlier (*15*) and more recent observations (*16*–*18*) in west and southwest Greenland, showing that basal water pressure is near ice-overburden pressure even close to the ice sheet margin late in the melt season, where the lowest pressure would be expected. The ice sheet elevation change in the region is on the order of 0.1 to 0.2 m a^−1^ over the period (*19*) because of a relatively high ice margin elevation of approximately 700 masl and the absence of large outlet glaciers, indicating a limited influence on catchment variations. The upper limit of the catchment varies from year to year and within a given season according to the elevation, termed the runoff limit, at which meltwater is generated in sufficient amounts to eventually reach the ice margin as runoff. A recent study (*20*) showed that surface meltwater channels in the extreme melt year 2012 reached up to ~1850 masl in the region, with 11 ± 4% of the runoff that season originating from above this elevation. An earlier study (*21*) showed that the mean equilibrium line altitude (where the surface mass balance is zero) over the period 1991–2011 was ~1550 masl, ranging from ~1240 to ~1830 masl.

**Fig. 2:**
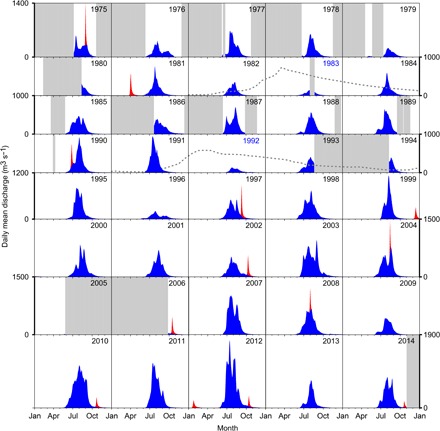
Seasonal discharge from the Tasersiaq catchment. Daily mean discharge (in blue) with GLOFs (in red) and missing data (gray boxes). The vertical scale (discharge) is similar for all plots but varies in extent according to the peak discharge of the 5-year period in question. Years with severe influence from volcanic eruptions are labeled in blue text, with the relative impact on lower stratospheric mean aerosol optical depth at 66.5°N over time shown as a dotted gray line (see Fig. 3).

A common feature of catchments adjoining the Greenland ice sheet is the occurrence of glacial lake outburst floods (GLOFs), which occur when the water volume stored in an ice-dammed lake becomes sufficient to lift the ice barrier blocking its path downstream. We identified 15 GLOFs originating from a single source lake (marked A in Fig. 1 and fig. S1) (see Materials and Methods and fig. S5). Because the typical interval between GLOFs is on the order of several years, they introduce noise in the relationship between annual discharge and climate forcing. Consequently, we chose to discard the additional water released to the larger catchment during the GLOFs by linear interpolation to cut off the peak in discharge time series between onset and end of each GLOF, as determined by the daily discharge rate of change (fig. S3). The result is a detailed 40-year time series of daily mean discharge as annual hydrographs, with observational gaps and identified peaks from GLOFs (Fig. 2).

**Fig. 3:**
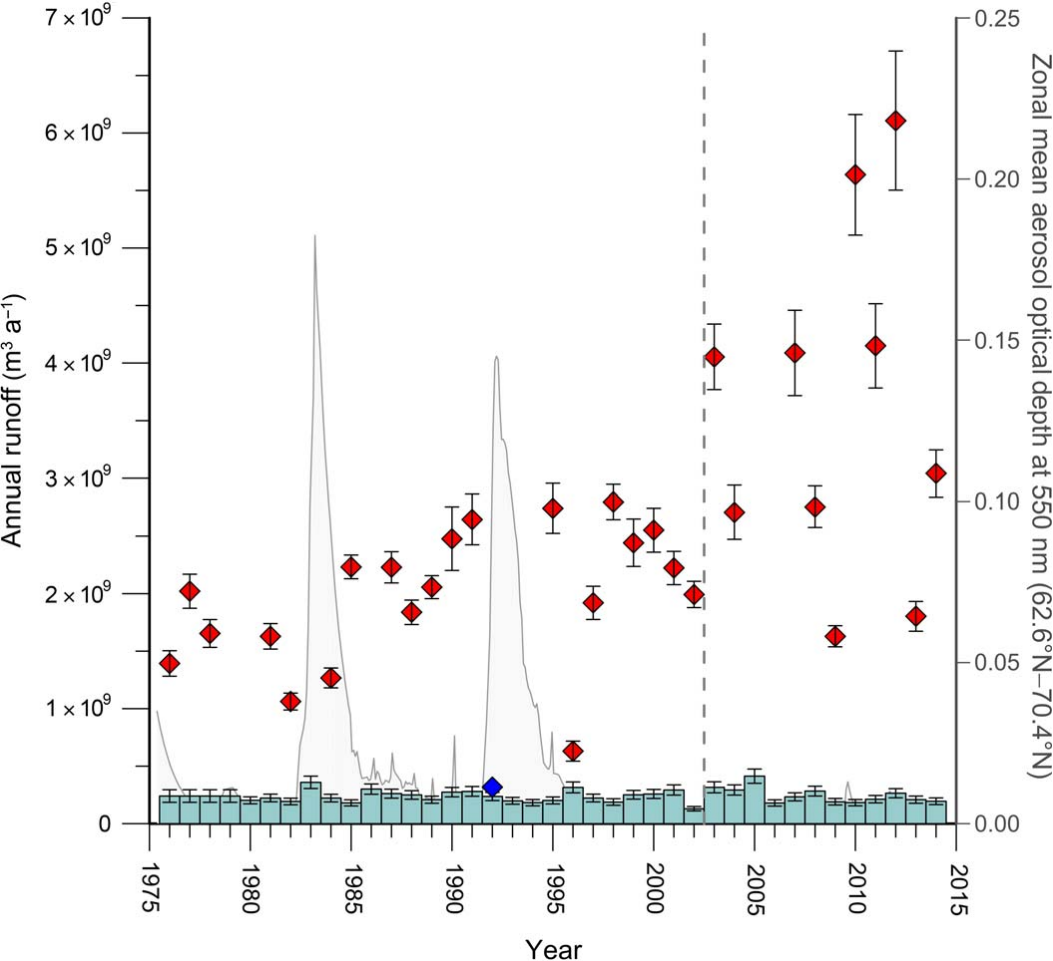
Annual ice sheet runoff from the Tasersiaq catchment. The annual runoff from the ice-covered part of the Tasersiaq catchment (left-side *y* axis). Red, data from regular years; blue, data from volcano-influenced year (only 1992 has sufficient data coverage; see Fig. 2); light blue bars, runoff from the ice-free part of the catchment derived from HIRHAM5, with uncertainties estimated from comparison to winter (September to May) surface mass balance measurements on the Amitsulôq ice cap (marked B in Fig. 1 and fig. S1) (*44*). Thin gray curve (right-side *y* axis): zonal mean aerosol optical depth at 550 nm at 66.5°N, indicating the attenuation of the sunlight from the aerosols as it passes through the atmosphere (*27*). The aerosol optical depth shows distinct peaks in 1983 and 1992 after major volcanic eruptions. The dashed vertical line indicates timing of the hypothesized change in runoff regime.

To obtain a time series of annual runoff from the ice sheet margin from the discharge time series, we first discarded years with insufficient data coverage, then removed the part of the signal because of GLOFs, and finally subtracted runoff originating from the ice-free part of the catchment, estimated by the regional climate model HIRHAM5 (see the Supplementary Materials) (*22*, *23*). To assess from which years we could derive annual runoff, we determined the extent of the melt season in the discharge time series to be between days 150 and 292 of the year, respectively. Using these limits, 31 years of annual ice sheet runoff remain out of the 40-year series (Fig. 3). We find that the six highest discharge years have occurred since 2003. Both the hydrographs and the annual ice sheet runoff time series exhibit clear impacts of the volcanic eruptions of El Chichón in Mexico in March to April 1982 and Mount Pinatubo in the Philippines in June 1991. Both eruptions injected sulfuric aerosol into the lower stratosphere, shading Earth’s surface, affecting global climate in the following 1 to 2 years (*24*, *25*), and reduced ice sheet surface melt in Greenland (*26*). Using the mean aerosol optical depth of the lower stratosphere at 66.5°N (Fig. 2) (*27*) as an indicator, we point out the catchment ice sheet runoff in 1983 and 1992 as severely influenced by stratospheric aerosols of volcanic origin, with a somewhat lesser impact in 1982, 1984, and 1993. Excluding the volcano-affected years 1983 and 1992, we find an abrupt change in the runoff regime occurring between 2002 and 2003 (Fig. 3) to be significant in a Rodionov climate regime shift test (*28*). The test criteria are fulfilled by transforming the catchment ice sheet runoff (*Q*). Applying the Rodionov regime shift test on ln(*Q*), we confirm a significant (*P* = 0.003) regime shift in 2003 from a mean of 2.0 km^3^ a^−1^ (SD, 0.57 km^3^ a^−1^) during 1976–2002 to a mean of 3.6 km^3^ a^−1^ (SD, 1.4 km^3^ a^−1^) during 2003–2014 (see Materials and Methods).

**Fig. 4:**
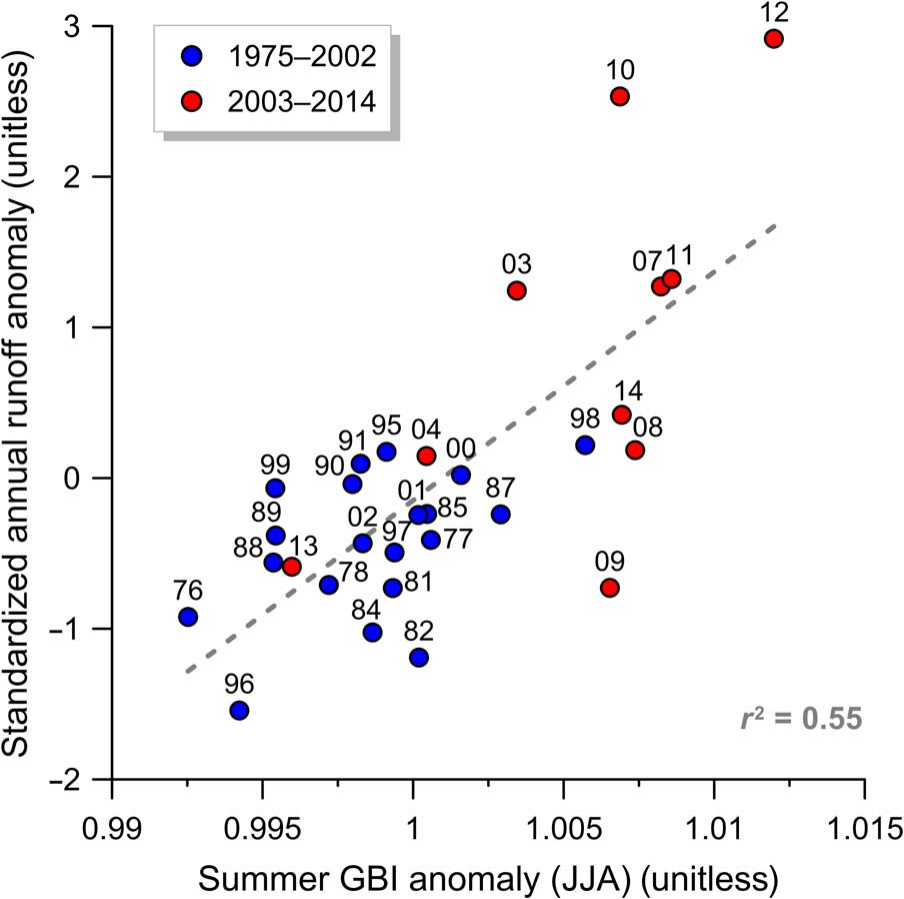
Relation between runoff and the GBI. The standardized annual runoff anomaly as a function of the summertime [June, July, and August (JJA)] GBI anomaly (volcano-affected years 1983 and 1992 excluded), where GBI is defined as the mean 500-hPa geopotential height between 60° to 80°N and 20° to 80°W (*30*). Blue, 2002 and before; red, 2003 and after. Labels within plot provide the last two digits of the year. Dashed line shows linear fit of blue and red entries with *r*^2^ = 0.55.

Greenland ice sheet surface melt intensity has been linked to persistent atmospheric circulation anomalies over Greenland associated with North Atlantic Oscillation negative phases (*29*). Specifically, the Greenland Blocking Index (GBI) defined as the mean 500-hPa geopotential height between 60° to 80°N and 20° to 80°W (*30*) has been strongly connected to surface melt, providing a metric for sustained anticyclonic flow, which favors west Greenland heating (*31*, *32*). Comparing mean summer (June to August) GBI with the standardized melt season runoff anomaly, the Tasersiaq runoff data confirm (*R*^2^ = 0.55, *P* < 0.001) GBI as a predictor of ice sheet surface melt (Fig. 4). The claim of a climate regime shift manifested as a change in the atmospheric circulation pattern is supported by most of the positive GBI anomalies occurring from 2003 and onward, with 2013 as the only notable exception. Although a positive GBI anomaly appears to be a necessary condition for extreme runoff, it is not in itself adequately characterizing the post-2003 period, as exemplified by comparing 2009 and 2010, which both have high GBI, yet widely varying runoff (Figs. 3 and 4).

To investigate possible changes in the origin of the summertime (JJA) air masses arriving at Tasersiaq, we performed an atmospheric trajectory analysis. The trajectories of air parcels were back-simulated using the Hybrid Single-Particle Lagrangian Integrated Trajectory model (HYSPLIT4) (*33*) and the 1975–2014 National Center for Atmospheric Research/National Centers for Environmental Prediction reanalysis data obtained from the National Oceanic and Atmospheric Administration, Air Resources Laboratory. All trajectories end within the Tasersiaq catchment at coordinates 66.25°N, 49.00°W and at an elevation of 5.5 kmasl, taken as representative of the 500-hPa geopotential surface elevation, which also forms the basis of the GBI calculation and is well suited to illustrate changes in general circulation patterns. From this end point, the simulation was run every third hour for 1 week back in time with a time step of 1 hour. The HYSPLIT4 model was set to use its default 1° × 1° terrain elevation model, with vertical motion calculated from the meteorological model’s vertical velocity fields and a model top at 10 km.

We mapped the resulting air parcel trajectory paths on a grid as the sum of the number of nodes in each grid cell, convolved with a bidimensional Gaussian kernel having a σ of 500 km, where a node corresponds to the position of an air parcel at hourly intervals along a trajectory. This value of σ was chosen empirically as a compromise between retaining large-scale spatial variability over the Arctic Ocean and its encircling land masses, and smoothing the fine detail of only local significance.

**Fig. 5:**
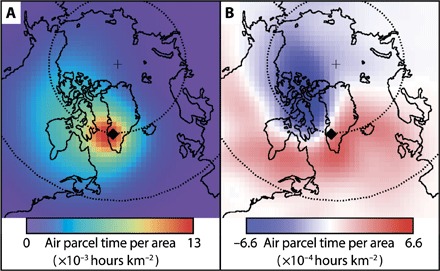
Atmospheric circulation change from trajectory path analysis. The origin (**A**) and change in origin (**B**) of summertime air masses at Tasersiaq. (A) The mean air parcel trajectory density for each summer (JJA), averaged over the entire period 1975–2014. The unit “air parcel time per area” denotes the time an air parcel eventually arriving at Tasersiaq has spent over a given area over the week before its arrival, based on modeled trajectory paths. (B) The difference between the mean air parcel trajectory density anomalies of the periods 1975–2002 and 2003–2014, illustrating a general shift toward the south in the origin and path of summertime air masses arriving in Tasersiaq.

This produced a gridded map of air parcel trajectory density for each summer (JJA), shown as an average over the entire period 1975–2014 in Fig. 5A. To investigate the change from year to year, we calculated the annual anomaly from the 1975–2014 mean air parcel trajectory density (shown in fig. S6). Subsequently, we compared the mean air parcel trajectory density anomaly between the periods 1975–2002 and 2003–2014 by subtracting the latter from the former (Fig. 5B). The resulting map illustrates the change in the origin of air masses arriving at Tasersiaq at 5.5 kmasl, that is, approximately at the altitude of the 500-hPa surface forming the basis of the GBI. The regime shift detected in the runoff time series after 2003 seems to coincide with a change in the origin of the air masses from higher latitudes (north of ca. 62°N) west of Greenland to a more southerly area covering ca. 45° to 62°N on the western side of Greenland and ca. 45° to 79°N on the eastern side (Fig. 5B).

We interpret this shift in the origin of the air masses arriving in southwest Greenland as supporting recent evidence (*34*) that the persistence and direction of the meridional flow along western Greenland determine the magnitude of the runoff in the post-2003 period (fig. S6), providing the northward advection of heat and moisture associated with recent years of extreme runoff (*35*, *36*).

## DISCUSSION

Although other time series of runoff from extensive catchments of the Greenland ice sheet exist (*37*), none has a temporal extent spanning 40 years, making this data set ideal for validation of modeled runoff from regional climate models (*4*–*8*). We argue that quantifying the impact of global warming on the Greenland ice sheet, as manifested through Arctic amplification and its effect on atmospheric circulation, requires time series exceeding the 30-year period needed to assess its climatological state.

The daily resolution of the runoff time series enables targeted validation of model performance, for example, surface routing, retention of meltwater/rain, and the ability to capture peak melt events. The integrated nature of the catchment scale runoff is ideally combined with model validation against ice sheet weather station data of individual climatological parameters, offering a combined evaluation of both the model biases and their impact on the modeled runoff (*38*). Although it would be useful with more gauged basins around the ice sheet to capture regional differences, only the south and southwestern parts of Greenland offer the ideal combination of extensive hydrological catchments and significant runoff. In terms of the southwestern Greenland ice sheet, the representative nature of the Tasersiaq catchment is supported by a comparison with eight overlapping years of discharge for the catchment of Watson River as observed at Kangerlussuaq (see Fig. 1) (*37*), approximately 100 km north of Tasersiaq, yielding a correlation of *R*^2^ = 0.95.

Increased frequency in blocking events and meridional flow has been linked to a wavier jet stream because of a reduced poleward temperature gradient, in turn an effect of the Arctic amplification of global warming (*39*). Here, we present observational evidence of an abrupt change in the runoff regime in southwest Greenland occurring in 2003, with an 80% increase in ice sheet runoff between the 1976–2002 and 2003–2014 periods, and link this to an increase in persistent summertime anticyclonic flow over Greenland through correlation with the GBI and a southward shift in the origin of the air masses arriving in the Tasersiaq catchment in southwest Greenland. We consider it likely that this change in the runoff regime driven by atmospheric changes could be further reinforced by the growth of ice layers in the firn of the lower accumulation area of the ice sheet, reducing meltwater retention (*20*).

## MATERIALS AND METHODS

### Measuring water level

The time series of discharge from lake Tasersiaq was calculated from a measured time series of water level in the lake and a stage-discharge relation. Continuous monitoring of lake Tasersiaq started in July 1975 with the establishment of the “Hydrometric Station 308” near the outlet of the lake. Positions of measuring stations and years of operation are given in table S1. In August 1978, the measuring equipment was upgraded and the measuring station was moved approximately 200 m along the lake shore away from the outlet. This station was named “Station 105-1.” Further minor relocations of the station and associated updates of the station name took place in 1994 and 2013. The water level was measured by pressure transducers placed at the lake bottom. At Station 308, a pressure point at the lake bottom was connected to a mercury column manometer on land. A float and paper chart registered changes in the position of the mercury surface. Daily water levels were manually read from the chart and later digitized. At all the other stations, water level was, and is, measured using electronic pressure sensors and data loggers. The cable connecting the pressure transducer with the data logger on land also contains a thin tube, enabling the sensor to compensate air pressure changes. For the 105-*x* stations, instantaneous measurement of water level has been stored year round by data logger every third hour. Since 1980, two pressure transducers have been connected to the 105-*x* stations, reducing the risk of data gap and increasing information to assess measurement uncertainties, for example, in case one sensor is moved by ice during spring breakup or similar events. On the basis of the water surface measurements made during site visits by optical leveling relative to reference points on land and concurrent measurements from the pressure transducers, the position of each sensor has been calculated. Changes in sensor position between visits have been investigated, and standard corrections for any sudden changes have been made (*40*). A time series of the position of the water surface was consequently calculated on the basis of the corrected sensor position and the measured water pressure.

### Establishing a stage-discharge relation

To establish a stage-discharge relation, we performed manual discharge measurements at a cross-section of the outlet river immediately downstream of the lake and station. All measurements were of the type where the velocity of the water was measured at selected depths in a number of verticals evenly distributed over a well-defined cross-section (*41*). Where the water depth allowed, the water velocity was measured at five or six depths in each vertical. At verticals with low water depth near the river edges, the water velocity was measured in a reduced number of points. The number of verticals was typical between 15 and 30, depending on the discharge during the time of the measurement. The discharge was then calculated by integration of the velocities over the cross-sectional area. The water velocities were measured by propeller-type current meters, either Nerflux from Neyrpic or Universalflügel (C-31) from OTT. A total of 40 manual discharge measurements were carried out. A stage-discharge relation was established in 1986 based on the 37 measurements, which were carried out by then. Since then, three measurements have been carried out: in 1986, 1989, and 2005. These three measurements are in good agreement with the established stage-discharge relation, indicating a stable section control at the outlet of the lake (fig. S2). With 40 measurements, the stage-discharge relation for Tasersiaq was well supported within the span of water levels of the manual discharge measurements. However, because a stage-discharge relation is an empirical relation, extrapolation beyond this interval has a higher degree of uncertainty. For the full discharge time series, 6.7% of the total discharge amount was found by extrapolation. However, for half of the years, the highest measured water level did not exceed the highest water level at which the discharge has been measured manually, and thus, for these years, extrapolation was not needed at all. Approximately 51% of data points have discharge values found by extrapolation to values below the minimum manually measured discharge of 7 m^3^ s^−1^. However, these low discharge values only contributed around 1% of the average annual discharge totals.

During winter, ice forming on the banks and the surface of the outlet river can change the resistance and cross-sectional area, in which case the shore ice–free stage-discharge relations may be invalid. Manual discharge measurements have not been carried out during winter at Tasersiaq, but measurements at other lake outlets in Greenland during winter showed only small deviation from the relevant stage-discharge relation (*42*).

Tasersiaq receives discharge from an ice-dammed lake in the form of outburst floods (Jökulhlaup or GLOF). These GLOFs also occur during winter. When the inflow of water from the ice-dammed lake ceases, the following decrease in water level in Tasersiaq is in good agreement with the recession curve calculated from the stage-discharge relation. This further supports that the deviation from the stage-discharge relation in winter is limited.

The uncertainty in the daily mean discharge (95% confidence level) was calculated, taking into account the uncertainty of the stage-discharge relation as well as the measurement uncertainty of the pressure transducers and the uncertainty in the determination of sensor positions (*43*). The additional contribution to the uncertainty in the annual discharge from filling of minor gaps in the discharge time series was estimated assuming an uncertainty during these periods of twice the mean uncertainty for that year, whereas the contribution to uncertainty from extrapolation of the stage-discharge relation during periods of very high flow was assumed to be three times the mean uncertainty for that year.

### Identifying GLOFs

We attributed the GLOFs to a single source lake (marked A in Fig. 1 and fig. S1) by positive identification of visible signs of lake drainage in aerial photos and satellite imagery during identified periods of sudden peaks (fig. S5). From this lake, 13 GLOFs were identified by examination of the rate of change in discharge, which exhibits a characteristic steep increase and subsequent decrease in response to the sudden inflow from the source lake (fig. S3). Two additional GLOFs occurring during periods with no discharge data were found by combining visible evidence with the number of positive degree-days since last GLOF (a proxy for meltwater inflow to the source lake of the GLOFs). The identified GLOF duration was 4 to 17 days (median, 6 days), with a progressively decreasing time interval and volume, indicating increasing melt rates and a thinning glacier tongue barrier (coordinates 66.16°N, 50.98°W).

### Testing for climate regime shift

The Rodionov climate regime shift test was conducted using the Sequential Regime Shift Detector (SRSD) software package version 5.1 for test of shifts in mean (*28*). The SRSD is available online at https://sites.google.com/site/climatelogic/home (accessed on 15 June 2017). The method requires a normally distributed, independent data set. These criteria were fulfilled by the natural logarithm of the catchment ice sheet runoff, ln(*Q*), as was shown by Shapiro-Wilks test and autocorrelations test, respectively. The magnitude and scale of the regimes to be detected by the SRSD were controlled by two input parameters: the target significance level and the cutoff length. The target significance level is the level at which the null hypothesis that the mean values of the two regimes are equal is tested using the two-tailed Student’s *t* test. The lower the significance level, the larger the magnitude of the shift should be to be detected. The cutoff length determines the minimum length of the regimes that are certain to be detected. If the regimes are shorter than the cutoff length, the probability for them to be detected reduces proportionally to their length. In a sensitivity analysis, all combinations of these two parameters identified one shift in the mean. For medium cutoff lengths (7 to 15 years) and significance level of 0.05 to 0.1, a shift in 2003 was detected (*P* = 0.003), whereas shorter cutoff lengths (5 to 7 years) indicated a less significant shift in 2010 (*P* = 0.048). Huber’s weight parameter was set to 3, giving equal weight to all values when calculating the regime means.

## Supplementary Material

http://advances.sciencemag.org/cgi/content/full/3/12/e1701169/DC1

## SUPPLEMENTARY MATERIALS

Supplementary material for this article is available at http://advances.sciencemag.org/cgi/content/full/3/12/e1701169/DC1 Catchment delineation The HIRHAM5 regional climate model experiment fig. S1. Outlet region of the Tasersiaq catchment. fig. S2. Stage-discharge relation for Tasersiaq. fig. S3. Signature rate of change of the discharge during a GLOF. fig. S4. Comparison between modeled and measured snow accumulation. fig. S5. Positive identification of the source lake of the GLOFs. fig. S6. The change in origin of summertime air masses at Tasersiaq. table S1. Position of measuring stations. References (*45*–*47*)
