# Supplementary material for: Abrupt shift in the observed runoff from the southwestern Greenland ice sheet
Source: Sci Adv. 2017 Dec 13;3(12):e1701169. doi: 10.1126/sciadv.1701169 (PMC5729017; doi:10.1126/sciadv.1701169)
Supplement: http://advances.sciencemag.org/cgi/content/full/3/12/e1701169/DC1 [file supp_3_12_e1701169__index.html]

Science Advances | Science Advances

## Supplementary Materials

**This PDF file includes:**

- Catchment delineation
- The HIRHAM5 regional climate model experiment
- fig. S1. Outlet region of the Tasersiaq catchment.
- fig. S2. Stage-discharge relation for Tasersiaq.
- fig. S3. Signature rate of change of the discharge during a GLOF.
- fig. S4. Comparison between modeled and measured snow accumulation.
- fig. S5. Positive identification of the source lake of the GLOFs.
- fig. S6. The change in origin of summertime air masses at Tasersiaq.
- table S1. Position of measuring stations.
- References (*45–47*)

Download PDF

**Files in this Data Supplement:**

- Adobe PDF - 1701169\_SM.pdf
