# Supplementary material for: Abrupt shift in the observed runoff from the southwestern Greenland ice sheet
Source: Sci Adv. 2017 Dec 13;3(12):e1701169. doi: 10.1126/sciadv.1701169 (PMC5729017; doi:10.1126/sciadv.1701169)
Supplement: http://advances.sciencemag.org/cgi/content/full/3/12/e1701169/DC1 [file 1701169_SM.pdf]

Science Advances

AAAS

advances.sciencemag.org/cgi/content/full/3/12/e1701169/DC1

Supplementary Materials for

Abrupt shift in the observed runoff from the southwestern Greenland ice sheet

Andreas P. Ahlstrøm, Dorthe Petersen, Peter L. Langen, Michele Citterio, Jason E. Box

Published 13 December 2017, *Sci. Adv.* **3**, e1701169 (2017)
DOI: 10.1126/sciadv.1701169

This PDF file includes:

- Catchment delineation
- The HIRHAM5 regional climate model experiment
- fig. S1. Outlet region of the Tasersiaq catchment.
- fig. S2. Stage-discharge relation for Tasersiaq.
- fig. S3. Signature rate of change of the discharge during a GLOF.
- fig. S4. Comparison between modeled and measured snow accumulation.
- fig. S5. Positive identification of the source lake of the GLOFs.
- fig. S6. The change in origin of summertime air masses at Tasersiaq.
- table S1. Position of measuring stations.
- References (*45*–*47*)

SUPPLEMENTARY MATERIALS

Catchment delineation

The delineation of the ice-free part of the catchment was straightforward and simplified by the lack of change in ice sheet extent over the study period 1975–2014, as evidenced by detailed orthophotos from 1985 and subsequent satellite images from the Landsat missions. However, the ice-covered parts introduced two challenges: 1) A *c*. 84 km^2 lake named "860" situated at 800 m a.s.l. lies adjacent to the ice sheet margin immediately south of Tasersiaq and has no visible outlet, making it a candidate for glacial lake outburst floods. Lake 860 is bordered by ice fronts along nearly 40% of its perimeter including the potential route away from the Tasersiaq catchment. To test whether Lake 860 was part of the Tasersiaq catchment, we calculated the water balance of the lake during two measurement periods, Oct. 5–23, 1977, and Sept. 9–28, 1980, when the water level was recorded during lake drainage events. During these periods, the water volume released from Lake 860 exceeded the inflow of water to Tasersiaq by a factor of 50 and 10, respectively, proving beyond any conceivable measurement uncertainty that it drains towards the south underneath the ice cover and is not part of the Tasersiaq catchment. 2) Meltwater on the ice sheet tends to drain into moulins and crevasses, entering the subglacial hydrological system rather than staying on the surface. The large-scale meltwater drainage is thus likely governed mainly by the basal water pressure field. A previous attempt at catchment delineation in this region (*14*) showed that changes in the underlying data coverage of basal topography can introduce large uncertainties in the delineation. A similar analysis utilizing the most recent dataset for the basal topography (*13*) which includes the same airborne ice-penetrating radar data as used in (*14*) complemented with topography estimated from inversion of ice velocity maps using mass-conservation principles, shows that this conclusion remains the

same. The current data coverage and resolution of basal topography must be considered as insufficient for this type of catchment delineation.

Another important quantity in the catchment delineation is the water pressure at the base of the ice sheet. Recent observations (*16–18*) in West and Southwest Greenland confirm early evidence that ice sheet basal water pressure is close to ice overburden pressure even close to the ice sheet margin late in the melt season, where the lowest pressure would be expected (*15*). One study (*17*) measured basal water pressure in boreholes along a transect with ice thickness ranging from 100 m to more than 800 m. All the 10 boreholes away from the terminus (17 km to 34 km) exhibited basal water pressure levels above 82% of ice overburden pressure throughout the melt season. Further away from the ice margin, even higher basal water pressure would be expected due to a less efficient subglacial drainage system. This range and distribution of basal water pressure points at a limited influence on the catchment delineation. On the scale of the Tasersiaq catchment, the surface elevation can thus be regarded as a reasonable first approximation of the height of the water pressure potential in the ice sheet subglacial drainage system. The Greenland ice sheet has experienced large changes in surface elevation which could influence the catchment delineation. However, the ice sheet elevation in the region has remained relatively stable with a modest change on the order of 0.1–0.2 m a-1 over the period (*19*). We attribute this to a relatively high ice margin elevation of approx. 700 m above sea level and notably an absence of large outlet glaciers affected by ocean changes. With a relatively stable geometry and a limited variation in basal water pressure, we expect catchment variations to be minor over the study period 1975–2014.

The HIRHAM5 regional climate model experiment

To assess the changes in the ice sheet runoff regime, we subtracted discharge from the ice-free part of the catchment from the ice sheet discharge. This was estimated using 6-hourly output from the HIRHAM5 regional climate model, run with the ERA-Interim reanalysis dataset (*22*) as input at the domain boundaries from 1980–2014 at a 5.5 km resolution (*23*). The HIRHAM5 regional climate model is developed by the Danish Meteorological Institute and the Potsdam Research Unit of the Alfred Wegener Institute Foundation for Polar and Marine Research. It combines the dynamics of the HIRLAM weather forecast model (*45*) with the physical parameterization schemes of the ECHAM climate model (*46*). In the current configuration, HIRHAM5 is run over a Greenland-wide domain at 5.5-km resolution (0.05° x 0.05° on a rotated pole grid) (*23*). Six hourly inputs of horizontal wind vectors, temperature, and specific humidity from the ERA-Interim reanalysis dataset (*22*) are supplied at the domain boundaries at all atmospheric levels to compute the atmospheric circulation within the domain at 90 s time steps. The model was run over the period 1979–2014, but surface runoff data from the first year was discarded as land surface module spin up.

Grid cells have in the experiment been classified as ocean, non-glaciated land or glacier cells. The distinction between the latter two has been made based on a recent glacier extent map (*47*), setting all cells with fractional glacier coverage greater than 0.5 to glacier. For the purposes of this study, we are strictly interested in the non-glaciated land surface runoff, which is calculated from the rainfall, evaporation, sublimation, evapotranspiration and snowmelt using a bucket approach for soil moisture (*46*). To account for runoff deriving from the parts of the ice-free catchment that fall within cells that have glacier fraction greater than 0.5 (but less than 1), runoff

from neighboring all-ice-free cells was averaged to provide a number for that cell, which was finally multiplied by the ice-free fraction of the cell. Likewise, for ice-free cells that only partially fall within the catchment basin, the cell's total runoff was multiplied by the fraction of the cell within the catchment.

While reliable measurements of solid precipitation are difficult to obtain directly (e.g. using precipitation gauges), glaciological winter mass balance measurements consisting of snow depth probing combined with density profiles obtained from snow pits from an ice cap provides an alternative method. The Tasersiaq catchment is situated in a precipitation shadow and is one of the driest regions in Greenland (*9*). A large portion of the runoff from the ice-free part of the catchment can be assumed to come from winter snowfall, implying that a comparison of observed and modeled winter surface mass balance provides a reliable way of quantifying the overall model performance with respect to runoff from the ice-free part of the catchment. The uncertainty of the estimated runoff from the ice-free catchment was derived by comparing winter (Sep-May) surface mass balance (SMB) measurements from 16 stakes covering 1982-1990 on the Amitsuloq ice cap (*44*) (marked B in Figs. 1 and S1) with winter SMB values from HIRHAM5 over the same period. The observed winter SMB from the 16 stakes were averaged for each of the nine years (1982–1990) to minimize observational noise, providing a single annual observed average winter SMB from the Amitsuloq ice cap. This average winter SMB from stake observations was subsequently compared to an annual modeled winter SMB from HIRHAM5, constructed by bi-linearly interpolating the gridded winter SMB output to the 16 stake locations and subsequently averaging those for each of the nine years. On average, the model underestimates the winter SMB by 6%, and the annual values correlate well (R^2=0.72,

$p<0.005$) (fig. S3). The relative root mean square error is 12% and we consequently chose a conservative error estimate of 15% for the modeled 1980–2014 ice-free catchment runoff. Assuming that the ice-free catchment runoff is a log-normally distributed quantity, we added an uncertainty of approx. 8% for 1975–1979, corresponding to an error margin that would keep us inside the 95% limit of the 1980–2014 distribution (Fig. 3). The 6% underestimation in the modeled Amitsuloq winter SMB is in line with previous analyses (*23*) finding the annual mean precipitation in the 0.05 degree model (as employed here) to be within 10% of the observations at the Danish Meteorological Institute (DMI) weather station 4231 in Kangerlussuaq (see Fig. 1). The same study (*23*) also found that for coastal DMI weather stations as a whole, precipitation at the driest locations were slightly overestimated. This would indicate that the small contribution of the ice-free part of the catchment to average runoff and hence to inter-annual variability as derived from HIRHAM5 is unlikely to be significantly underestimated.

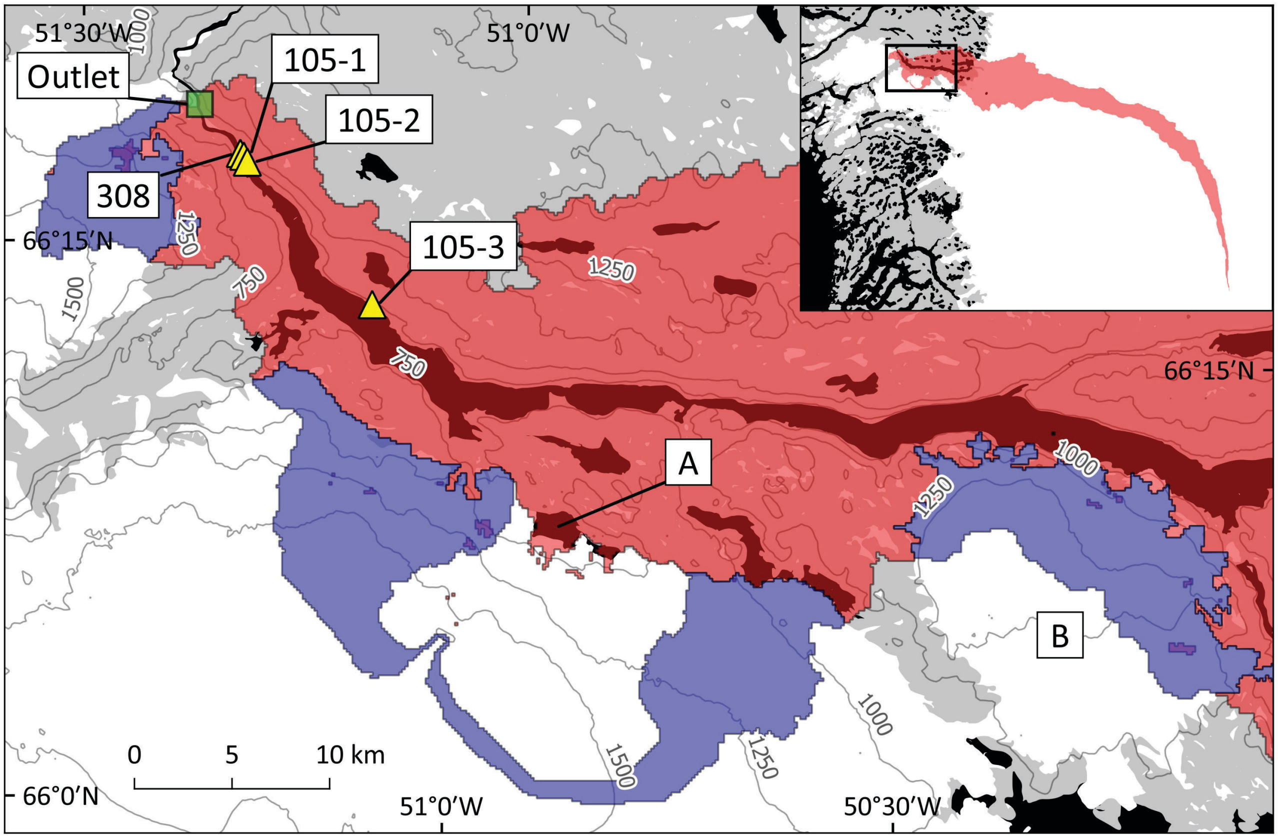

fig. S1. Outlet region of the Tasersiaq catchment. Location of stations 105-1, 105-2, 105-3 and 308 (yellow triangles) near the outlet (green square). Red: the ice-free part of the outlet region of the catchment, blue: the part of the catchment covered by local ice caps, A: an unnamed ice-dammed lake delivering glacial lake outburst floods to the catchment (subtracted from the discharge time series shown in Fig. 2), B: the Amitsuloq ice cap. Insert: map location in the catchment presented in Fig. 1 marked with a black box. Note that the local ice cap catchment to the ice-dammed lake marked A is masked out of the Tasersiaq catchment delineated in this study.

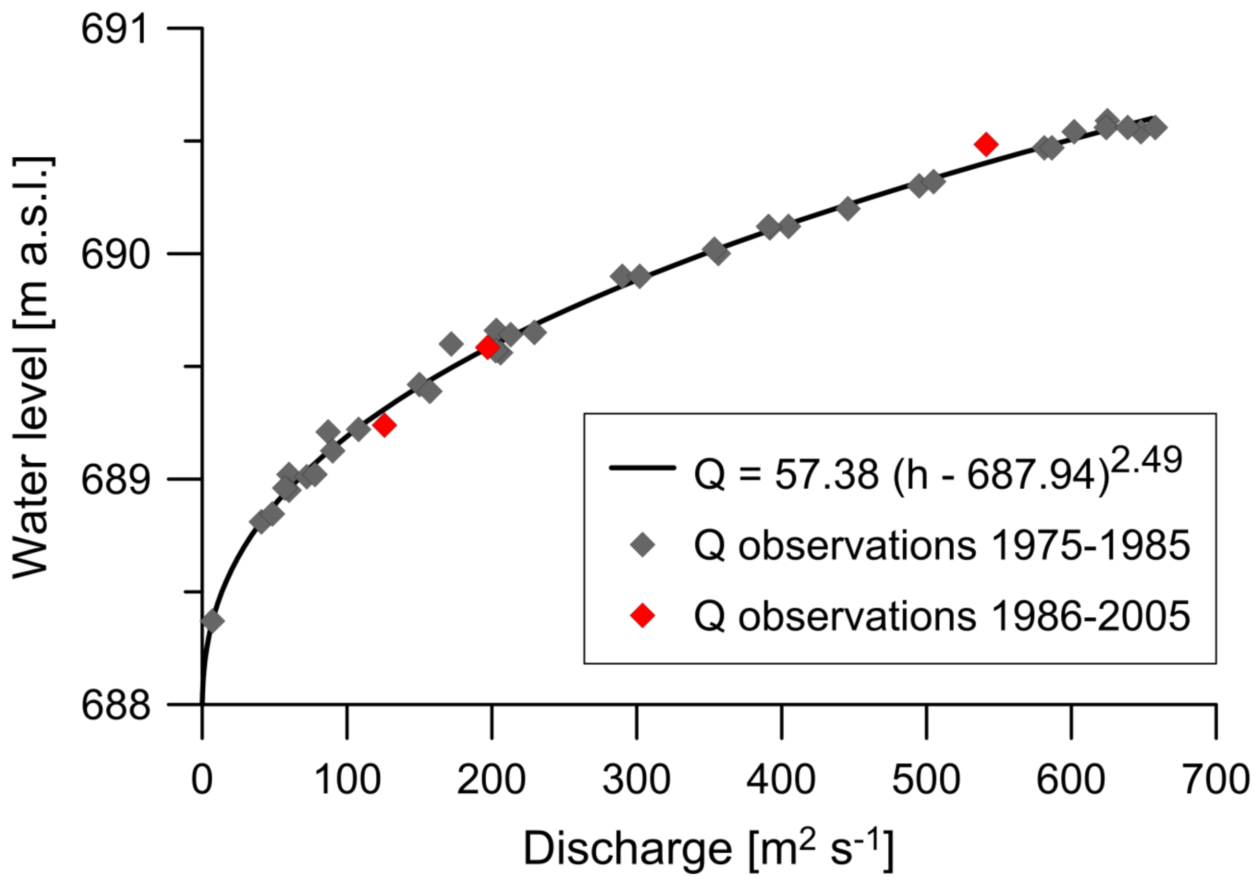

fig. S2. Stage-discharge relation for Tasersiaq. The relation between water level and discharge for Tasersiaq, based on 37 measurements carried out 1975–1985 and validated by 3 subsequent measurements in 1986, 1989 and 2005.

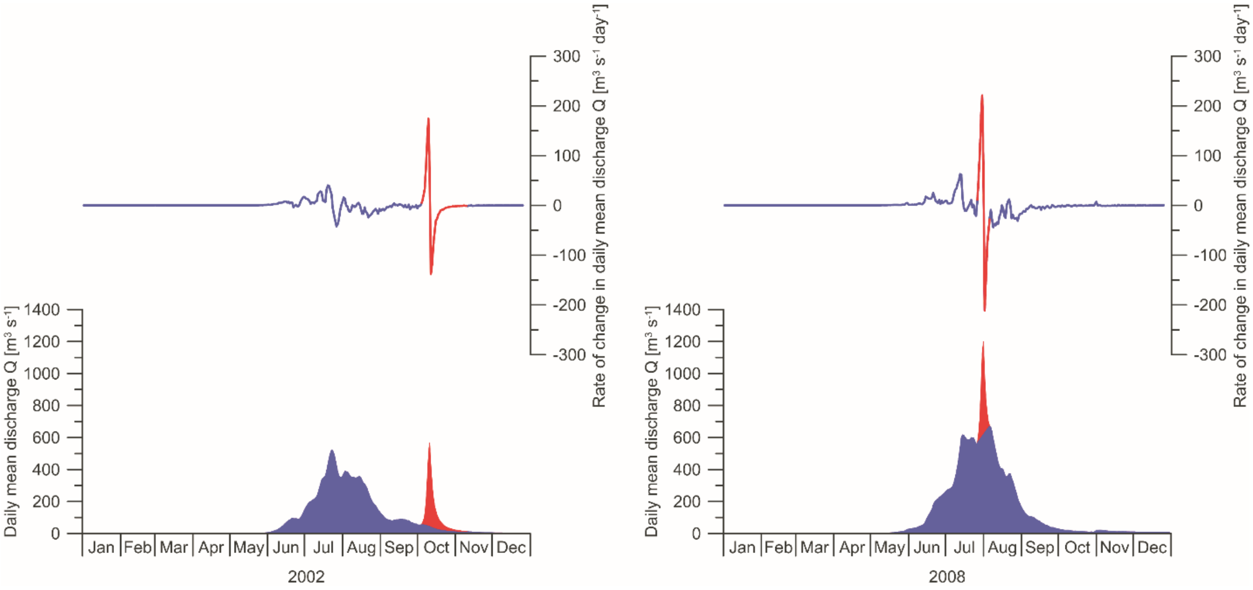

fig. S3. Signature rate of change of the discharge during a GLOF. The red colour shows the part identified as related to a glacial lake outburst flood, exhibiting a characteristic sharp rise and subsequent fall, allowing for positive identification even during the peak melt season as seen in the panel on the right. In total, 3.57 percent of the time series (less than 1.86 percent of the discharge) used for establishing annual discharge sums was derived from values determined by discarding peaks due to GLOFs.

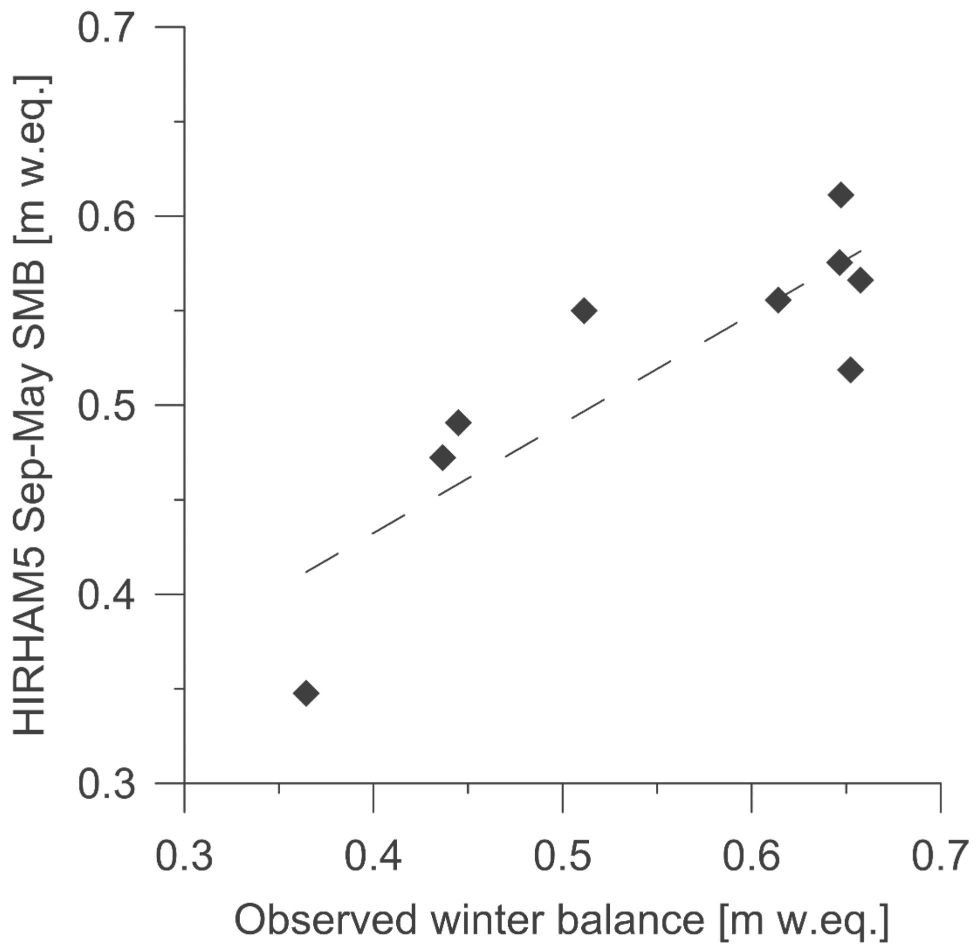

fig. S4. Comparison between modeled and measured snow accumulation. Winter (Sep–May) surface mass balance (SMB) measurements from 16 stakes covering 1982–1990 on the Amitsuloq ice cap (*44*) (marked B in Fig. 1 and fig. S1) plotted against bi-linearly interpolated winter SMB values from HIRHAM5 over the same period. For each of the nine years (1982–1990), an average was taken over the 16 sites for both observed and modeled balance, thus providing inter-comparable annual values. On average, the model underestimates the winter SMB by approx. 6%, and the annual values correlate with R^2=0.72 ($p<0.005$), with a relative root mean square error of 12%.

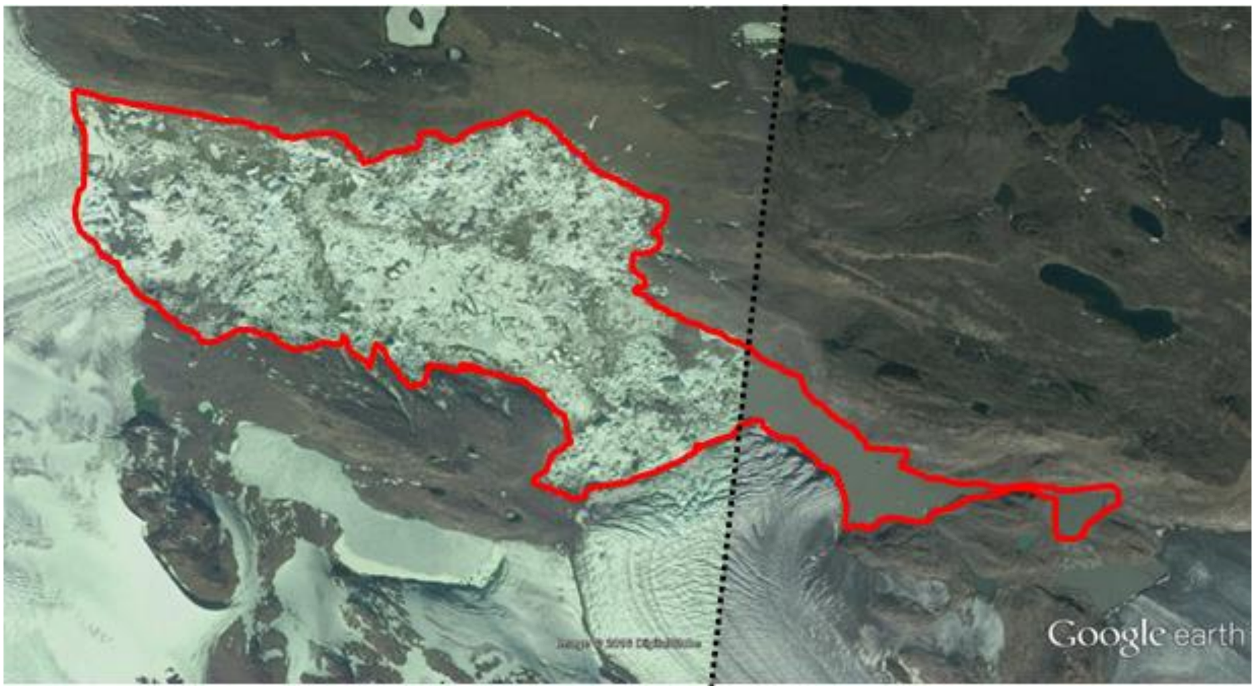

fig. S5. Positive identification of the source lake of the GLOFs. Close-up of a merged image consisting of two satellite images showing the source lake (outlined in red) for the glacial lake outburst floods (GLOF's), separated by the dotted line. The right/left part of the image shows the lake before/after a GLOF event (images are from June 22, 2011, and July 11, 2012, respectively). After the GLOF event, icebergs exhibit more contrast as they are exposed sitting on the bottom of the nearly empty lake (Image: Google Earth, DigitalGlobe).

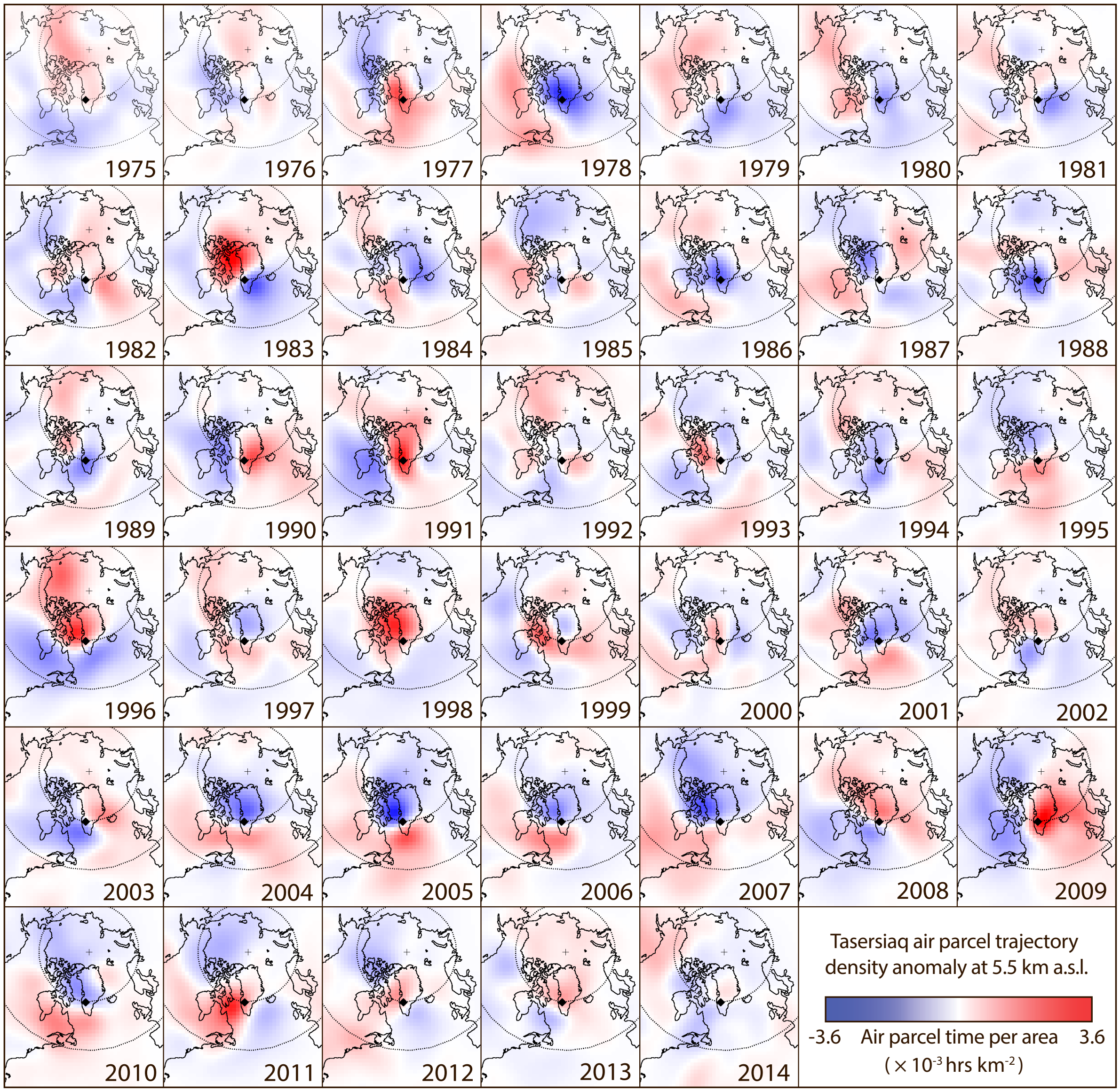

fig. S6. The change in origin of summertime air masses at Tasersiaq. The annual summertime (JJA) anomaly from the 1975–2014 mean air parcel trajectory density (shown in Fig. 5A) for air masses arriving at Tasersiaq (marked by a black diamond on the map) at an altitude of 5500 m a.s.l. The unit 'air parcel time per area' denotes the time an air parcel eventually arriving at Tasersiaq has spent over a given area over the week prior to its arrival, based on modeled trajectory paths.

table S1. Position of measuring stations. The number, position, elevation and operational period of the measuring stations operated by Asiaq near the outlet of the Tasersiaq catchment. The positions of the four stations in the catchment are marked in fig. S1.

Station no.	Longitude (W)	Latitude (N)	Elevation (m.a.s.l.)	Start date	End date
308	051˚18’22’’	66˚18’04’’	*c*. 690	July 7, 1975	Dec. 31, 1980
105-1	051˚18’07’’	66˚18’00’’	*c*. 730	Aug. 27, 1978	Aug. 20, 1993
105-2	051˚17’50’’	66˚17’51’’	*c*. 690	Aug. 10, 1994	July 28, 2014
105-3	051˚08’24’’	66˚14’22’’	*c*. 700	July 13, 2013	In operation
